# Temoporfin-Conjugated Upconversion Nanoparticles for NIR-Induced Photodynamic Therapy: Studies with Pancreatic Adenocarcinoma Cells In Vitro and In Vivo

**DOI:** 10.3390/pharmaceutics15122694

**Published:** 2023-11-28

**Authors:** Oleksandr Shapoval, David Větvička, Vitalii Patsula, Hana Engstová, Olga Kočková, Magdalena Konefał, Martina Kabešová, Daniel Horák

**Affiliations:** 1 Institute of Macromolecular Chemistry, Czech Academy of Sciences, Heyrovského nám. 2, 160 00 Prague, Czech Republic; 2 Institute of Biophysics and Informatics, First Faculty of Medicine, Charles University, Salmovská 1, 120 00 Prague, Czech Republic; 3 Institute of Physiology, Czech Academy of Sciences, 142 20 Prague, Czech Republic

**Keywords:** upconversion, temoporfin, photodynamic therapy, pancreatic tumor

## Abstract

Upconverting nanoparticles are interesting materials that have the potential for use in many applications ranging from solar energy harvesting to biosensing, light-triggered drug delivery, and photodynamic therapy (PDT). One of the main requirements for the particles is their surface modification, in our case using poly(methyl vinyl ether-*alt*-maleic acid) (PMVEMA) and temoporfin (THPC) photosensitizer to ensure the colloidal and chemical stability of the particles in aqueous media and the formation of singlet oxygen after NIR irradiation, respectively. Codoping of Fe^2+^, Yb^3+^, and Er^3+^ ions in the NaYF_4_ host induced upconversion emission of particles in the red region, which is dominant for achieving direct excitation of THPC. Novel monodisperse PMVEMA-coated upconversion NaYF_4_:Yb^3+^,Er^3+^,Fe^2+^ nanoparticles (UCNPs) with chemically bonded THPC were found to efficiently transfer energy and generate singlet oxygen. The cytotoxicity of the UCNPs was determined in the human pancreatic adenocarcinoma cell lines Capan-2, PANC-01, and PA-TU-8902. In vitro data demonstrated enhanced uptake of UCNP@PMVEMA-THPC particles by rat INS-1E insulinoma cells, followed by significant cell destruction after excitation with a 980 nm laser. Intratumoral administration of these nanoconjugates into a mouse model of human pancreatic adenocarcinoma caused extensive necrosis at the tumor site, followed by tumor suppression after NIR-induced PDT. In vitro and in vivo results thus suggest that this nanoconjugate is a promising candidate for NIR-induced PDT of cancer.

## 1. Introduction

Photodynamic therapy (PDT) is an effective clinical approach to the treatment of inoperable tumors that involves the interaction of a photosensitizer (PS) with visible light and oxygen to produce reactive oxygen species (ROS) that can damage biomolecules and kill cells [1]. Compared to conventional cancer treatments such as chemotherapy and radiotherapy, PDT has significantly fewer side effects due to its non-invasiveness, negligible drug resistance, and localized nature of treatment. PDT has been used to treat a variety of diseases, such as acne, age-related macular degeneration, and early-stage cancers, including skin, esophageal, mouth, and lung cancers [2,3]. Several types of PSs have been developed so far to achieve effective PDT, such as phthalocyanines [4], naphthalocyanines [5], porphyrins [6], chlorins [7], bacteriochlorins [8], pheophorbide [9], and texaphyrins [10,11]. Their efficacy depends on physicochemical properties, photostability, absorption coefficient, minimal dark toxicity in the absence of irradiation, localization of tumor, and efficiency of singlet oxygen (^1^O_2_) formation. Conventional PSs, when excited by light, emit radiation, usually in the UV or visible range (400–700 nm). In cancer treatment, they suffer from a relatively small depth of light penetration into biological tissues. To compensate for this limitation, PSs should be excitable with light in the near-infrared (NIR) region to penetrate deeper into tissues and consequently allow high spatial resolution and sensitivity [2,12,13]. Here, porphyrin- or chlorin-based PSs have the advantage over conventional sensitizers in terms of good purity, photosensitivity, tissue selectivity, and long-wavelength absorption with high ROS generation [14]. An example of a highly effective PDT agent is temoporfin (THPC; 5,10,15,20-tetra(m-hydroxyphenyl)chlorin; Foscan^®^) due to its unique biopharmaceutical properties and high singlet oxygen yield. THPC has been approved by the European Medical Agency for the palliative treatment of head and neck cancer and is also used in the treatment of breast, prostate, and pancreatic cancers [15,16]. THPC has enhanced light absorption in the red region (λ_max_ 652 nm) with a high molar extinction coefficient (2.9 × 10^4^ 1/cm∙M^−1^), which is higher than that of conventional porphyrins [17]. High ^1^O_2_ yields are achieved at a very low dose (0.15 mg/kg) and low energy intensity (10 J/cm^2^). However, the poor solubility of THPC, caused by the hydrophobicity of its aromatic macrocycles, leads to aggregation in aqueous media, resulting in fluorescence quenching and reduced ROS generation efficiency. In addition, THPC does not utilize the optimal biological spectral window for tissue penetration in the 700–1000 nm range. 

In recent decades, considerable efforts have been devoted to the development of upconversion nanoparticles, which have proven to be useful as nanotransducers [18]. Upconversion nanoparticles serve as an indirect excitation source to activate PSs in the NIR region, overcoming the limitations of molecular PSs such as hydrophobicity, non-specificity, and excitation in the UV-Vis region. Energy transfer from upconversion nanoparticles to PSs occurs via radiative (direct absorption of emitted PS photons) or non-radiative (Foster resonance energy transfer) mechanisms [19]. Due to the high upconversion efficiency with near-zero photobleaching, photoblinking, and background autofluorescence, Yb^3+^- and Er^3+^-doped upconversion nanoparticles have been extensively studied in PDT after NIR laser excitation (~980 nm), producing upconversion green (540 nm) and red (650 nm) luminescence to enable efficient bioimaging. The luminescence spectra overlapped with the absorption spectra of the most commonly used PSs, such as Rose Bengal [20], aluminum phthalocyanine [21], chlorin 6 [22], and methylene blue [23], resulting in ROS production and the killing of cancer cells. Moreover, Tm^3+^- and Yb^3+^-doped upconversion nanoparticles emitting in the blue region (450 and 475 nm) activated riboflavin [24], fullerenes [25], and temoporfin [26]. However, the low efficiency of energy transfer from upconversion nanoparticles to PSs due to the low intensity of red emission is still a challenge. 

**Figure 1:**
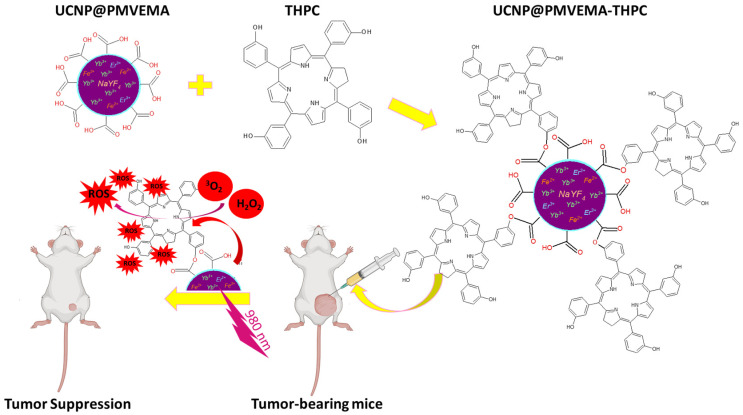
Schematic representation of the synthetic procedures of THPC-conjugated UCNP@PMVEMA particles and 980 nm NIR-induced PDT of pancreatic adenocarcinoma in an animal model.

In this study, we have for the first time designed and synthesized surface-engineered upconversion nanoparticles with covalently conjugated THPC, a clinically used PDT prodrug. THPC was bound to the surface of UCNP@PMVEMA particles by esterification and the Steglich reaction. Incorporation of Fe^2+^ ions into the particles was aimed at increasing the luminescence intensity in the red region for direct excitation of THPC. The cytotoxicity and photodynamic activity of the nanoparticles were studied in vitro and in a pilot in vivo therapeutic experiment of pancreatic adenocarcinoma in an animal model. This concept of NIR-induced PDT using UCNP@PMVEMA-THPC nanoconjugates is shown in Figure 1.

## 2. Experimental 

### 2.1. Materials 

*N*-(3-Dimethylaminopropyl)-*N*′-ethylcarbodiimide (EDC), 4-dimethylaminopyridine (DMAP), 2-(*N*-morpholino)ethanesulfonic acid (MES), 1,3-diphenylisobenzofuran (DPBF; 97%), RPMI 1640 medium, HEPES buffer ammonium fluoride (99.99%), octadec-1-ene (90%), phosphate-buffered saline (PBS), fetal bovine serum (FBS), Dulbecco’s modified Eagle medium (DMEM), and chlorides of yttrium (YCl_3_; 99%), erbium (ErCl_3_·6H_2_O; 99%), ytterbium (YbCl_3_; 99%), and iron (FeCl_2_∙4H_2_O) were purchased from Sigma-Aldrich (St. Louis, MO, USA). Poly(methyl vinyl ether-*alt*-maleic acid) (*M*_w_ = 60 kDa; PMVEMA; Figure S1a) was from Scientific Polymer Products (Ontario, NY, USA). Spectroscopic-grade dimethylsulfoxide (DMSO; 99.99%), hexane (99.5%), methanol (99.5%), sodium hydroxide, sodium chloride, perchloric, nitric, and oleic acids were purchased from Lach-Ner (Neratovice, Czech Republic). All other chemicals were from commercial sources and used without further purification. Rat insulinoma INS-1E cells (No. C0018009) were purchased from AddexBio (San Diego, CA, USA). Fetal calf serum was purchased from Life Technologies (Carlsbad, CA, USA). Other reagent-grade chemicals were obtained from commercial sources and used as received. Buffers and solutions were prepared from ultrapure water obtained by reverse osmosis with UV treatment (Milli-Q Gradient A10 system; Millipore; Molsheim, France). 5,10,15,20-tetra(m-hydroxyphenyl)chlorin (THPC; temoporfin; Foscan^®^; Figure S1b) was provided by MedChemExpress (Monmouth Junction, NJ, USA).

### 2.2. Synthesis of NaYF_4_:Yb^3+^,Er^3+^,Fe^2+^ Nanoparticles (UCNPs) 

UCNPs were prepared by the high-temperature coprecipitation method. Briefly, a 100 mL three-neck round-bottom flask was loaded with YCl_3_ (1.2 mmol), YbCl_3_ (0.4 mmol), ErCl_3_·6H_2_O (0.3 mmol), FeCl_2_·4H_2_O (0.1 mmol), octadec-1-ene (30 mL), and oleic acid (12 mL), and the mixture was heated to 170 °C, and stirred for 30 min with a magnetic stirrer under an argon atmosphere. The mixture was then cooled to room temperature (RT), a solution of NaOH and NH_4_F (5/8 mol/mol) in methanol (20 mL) was added dropwise, and the mixture was slowly heated to 70 °C to evaporate the methanol. Heating was continued at 300 °C for 1.5 h with stirring under an argon atmosphere. The resulting UCNPs were separated by centrifugation (3460 rcf) for 1 h, washed four times with a hexane/ethanol mixture (1/4 *v*/*v*), three times with ethanol, twice with ethanol/water (1:1 *v*/*v*), and ten times with water (14 mL each time), and redispersed in water. For physicochemical characterization, part of the dispersion was vacuum-dried at RT for 3 days. 

### 2.3. Modification of UCNPs with PMVEMA 

PMVEMA-coated UCNPs (UCNP@PMVEMA) were prepared according to the previously described procedure [27]. Briefly, PMVEMA (250 mg) was dissolved in water (5 mL) at RT, and the pH was adjusted to 7.4 using 2 M NaOH. The aqueous dispersion of UCNPs (10 mg UCNPs; 3 mL) was added dropwise to the PMVEMA solution with shaking at RT for 30 min and stirring at 70 °C for 12 h. The resulting UCNP@PMVEMA particles were washed three times with water using centrifugation (14,100 rcf) for 20 min to remove unbound PMVEMA and redispersed in water.

### 2.4. Conjugation of THPC to UCNP@PMVEMA Particles 

To exchange water for DMSO or MES buffer (pH 5.2), the aqueous UCNP@PMVEMA dispersion (2 mg/mL; 500 µL) was centrifuged (14,100 rcf) for 30 min, the supernatant was removed, and the particles were washed twice with DMSO or MES. Finally, the UCNP@PMVEMA particles were sonicated in DMSO or MES (2 mL) to a concentration of 1 mg/mL. 

#### 2.4.1. Conjugation Method I 

For surface activation, 100 µL of EDC (1 mg) and 200 µL of DMAP (1 mg) in 0.1 M MES buffer were added to a dispersion of UCNP@PMVEMA particles in 0.1 M MES buffer (1 mg/mL; 2 mL), and the mixture was stirred at RT for 30 min. A solution of THPC (0.6 mg) in DMSO (0.5 mL) was added, and the mixture was stirred at RT for 48 h in the dark, followed by centrifugation (12,000 rcf) for 20 min. The resulting UCNP@PMVEMA-THPC-1 particles were washed several times with ethanol and water and redispersed in water to the desired concentration. 

#### 2.4.2. Conjugation Method II 

The THPC-conjugated UCNPs were also prepared by modification of a procedure for porphyrin [28,29]. Briefly, NaOH pellets (20 mg) were added to a solution of THPC (1 mg) in DMSO (1 mL). The mixture was stirred for 30 min, and UCNP@PMVEMA dispersion in DMSO (1 mg/mL; 2 mL) was added dropwise over 10 min and kept at RT for 48 h with stirring under an argon atmosphere in the dark. Finally, the NaOH pellets were removed, and the conjugate, denoted as UCNP@PMVEMA-THPC-2, was separated by centrifugation (14,000 rcf) for 20 min, washed several times with ethanol and water, and redispersed there to the desired concentration. 

### 2.5. Characterization of UCNPs 

Particle morphology was examined by a Tecnai Spirit G2 transmission electron microscope (TEM; FEI; Brno, Czech Republic) [30]. The particle size distribution characterized by dispersity (*Ð* = *D*_w_/*D*_n_) was obtained by counting at least 300 particles from the micrographs to determine the number- (*D*_n_) and weight-average particle diameter (*D*_w_). The TEM microscope was equipped with an energy-dispersive X-ray (EDX) spectrometer (Mahwah, NJ, USA) used for analysis of the elemental composition of the nanoparticles. Hydrodynamic nanoparticle diameter (*D*_h_), polydispersity (*PD*), and *ξ*-potential were measured using dynamic light scattering (DLS; ZSU 5700 Zetasizer Ultra Instrument; Malvern Instruments; Malvern, UK) at 25 °C; *D*_h_ and *PD* were calculated from the intensity-weighted distribution function obtained by CONTIN analysis of the correlation function embedded in Malvern software (ZS XPLORER v. 1.3.2.27). Samples for elemental analysis (2–10 mg) were digested with HNO_3_ (0.3 mL) and HClO_4_ (1 mL) in a Biotage Initiator microwave reactor (Biotage; Uppsala, Sweden). After the digestion, the concentrations of Y^3+^, Er^3+^, and Yb^3+^ in the particles were determined with a NexION 2000B inductively coupled plasma mass spectrometer (PerkinElmer; Waltham, MA, USA); a standard solution (100 mg/L) of Y, Er, and Yb in 5% HNO_3_ with a concentration range of 0.04–0.17 μg/L was used for calibration. Fe^2+^ and Na^+^ contents were determined after the digestion using a Perkin Elmer 3110 atomic absorption spectrometer (PerkinElmer; Waltham, MA, USA) equipped with a hollow cathode lamp specific to each element. Thermogravimetric analysis (TGA) of particles was performed in air with a Perkin Elmer TGA 7 analyzer (Norwalk, CT, USA) over the temperature range 30–700 °C at a constant heating rate of 5 °C/min. FTIR spectra were recorded using a 100T FTIR spectrometer (Perkin-Elmer; Waltham, MA, USA) equipped with a Specac MKII Golden Gate single attenuated total reflection (ATR). X-ray diffraction (XRD) patterns of particles were taken with CuKα radiation (*λ* = 1.54 Å) using a high-resolution diffractometer GNR Explorer (Novara, Italy) with a Mythen 1K strip detector (STOE; Darmstadt, Germany). Measurements were performed in Bragg-Brentano geometry in the 2*Ө* range of 2–70° with a step of 0.05° and a time of 15 s at each step. The degree of crystallinity was calculated as the ratio of the crystalline part relative to the sum of the amorphous and crystalline parts of the spectra. The crystallite sizes were estimated according to the Scherrer equation *D*_XRD_ = K*·λ/*β·cos*Θ*, where *D*_XRD_ is crystallite size, K = 0.89 is the Scherrer constant, *λ* = 1.54 Å is the X-ray wavelength, β is a full width at peak half-maximum, and *Ө* is the Bragg angle [31]. Emission and excitation spectra were recorded on a FS5 Edinburgh Instruments spectrofluorometer (Edinburgh, UK) coupled with UV and 980 nm CW laser with 2 W output power (MDL-III-980). The content of temoporfin in the THPC-conjugated nanoparticles in water was determined by an Evolution 220 UV-Visible spectrophotometer (Thermo Fisher Scientific; Waltham, MA, USA) at 651 nm and compared to the calibration curve of THPC in ethanol. 

The generation of hydroxyl radicals by the Fenton reaction was determined spectrophotometrically using methylene blue. The UCNP@PMVEMA particle dispersion (0.2 mL; 2 mg/mL) in 0.1 M PBS or water was mixed with 3.5 mM methylene blue solution in water (2 mL) at RT in the dark, which was followed by the injection of 30% unstabilized H_2_O_2_ (0.5 mL). The time dependence of methylene blue degradation corresponding to the production of hydroxyl radicals was monitored by a Specord 250 Plus UV-Vis spectrophotometer (Analytik Jena; Jena, Germany) at 500–750 nm. 

Singlet oxygen generation was detected using a DPBF probe [32] on a Specord 250 Plus UV-Vis spectrophotometer (Analytik Jena). An aqueous dispersion of UCNP@PMVEMA-THPC (0.2 mL; 2 mg/mL) was added to a fresh solution of 10 mM DPBF in 50/50 (*v*/*v*) ethanol/water. The mixture was irradiated with a 980 nm laser (MDL-III-980-2W; 2.11 W/cm^2^) in the dark for various time intervals. The DPBF absorption intensity was monitored at 350–650 nm as a function of irradiation time, and the decrease in peak intensity at 415 nm was related to the formation of ^1^O_2_. 

### 2.6. Cytotoxicity Assay

The human pancreatic adenocarcinoma cell lines Capan-2, PANC-01, and PA-TU-8902 were purchased from the DSMZ-German collection of microorganisms and cell cultures (Leibniz Institute; Berlin, Germany). Cells were grown in culture flasks in DMEM supplemented with glucose (4.5 g/L), 1 mM sodium pyruvate, 1% penicillin/streptomycin (Gibco; Waltham, MA, USA), and 10% FBS for PANC-01 and PA-TU-8902 and 20% FBS for Capan-2 at 37 °C in a 5% CO_2_ atmosphere. Before experiments, cells were washed with PBS and incubated in 0.025% trypsin and 0.01% ethylenediaminetetraacetic acid in PBS for 5–10 min to achieve detachment. Cytotoxicity of PMVEMA-coated UCNPs with/without bound THPC was assessed by an XTT assay (Sigma-Aldrich) on three pancreatic carcinoma cell lines (Capan-2, PANC-01, and PA-TU-8902) according to the manufacturer’s protocol. Briefly, harvested cells were resuspended in growth medium and seeded into 200 µL 96-well plates (1 × 10^4^ cells per well). The following day, 50 µL of the tested UCNP dispersion was added to reach a concentration of 0.001–0.3 mg/mL, and the plates were incubated for 48 h in a CO_2_ incubator. After 48 h, 150 µL of supernatant was discarded and 25 µL of a mixture of sodium 3′-[1-(phenylaminocarbonyl)-3,4-tetrazolium]-bis(4-methoxy-6-nitro)benzene sulfonic acid hydrate (XTT) and phenazine methosulfate was added to the plates, and incubation continued for 2 h. The absorbance of the samples was measured at 450 nm with a reference wavelength of 620 nm using a Tecan Infinite^®^ F50 plate reader (Schoeller; Prague, Czech Republic).

### 2.7. In Vitro Photodynamic Activity 

To assess the photodynamic activity in vitro, rat insulinoma INS-1E cells were cultivated with 11 mM glucose in RPMI 1640 medium with *L*-glutamine supplemented with 10 mM HEPES, 1 mM pyruvate, 5% (*v*/*v*) fetal calf serum, 50 μmol/L mercaptoethanol, 50 IU/mL penicillin, and 50 μg/mL streptomycin; the cells (2 × 10^5^) were seeded on a poly(*L*-lysine)-coated cover slip two days before the experiment. The particle dispersion (0.3 mg/mL) was incubated with the cells for 20 h, excited at 980 nm by a Coherent 170 fs pulsed Chameleon laser with a power of 40 mW for 30 min, and observed using a Leica SP 8 confocal microscope (Leica Microsystems; Wetzlar, Germany) in bright field mode using the transmitted light detector. 

### 2.8. In Vivo Photodynamic Therapy

A total of 16 outbred nude female mice (Hsd: athymic Nude-Fox n1nu) of bodyweight between 19 and 23 g obtained from AnLab and ENVIGO (Prague, Czech Republic) were used in the study. The animals were kept in laminar flow cabinets with radiation-sterilized SAWI bedding (Jelu-Werk; Rosenberg, Germany), fed an irradiated Ssniff diet (Ssniff Spezialdiaeten; Soest, Germany), and had unlimited access to autoclaved water. All experiments were approved by the ethics committee of the First Faculty of Medicine, Charles University, and by the Ministry of Education, Youth, and Sports of the Czech Republic. Experiments were performed in accordance with Act No. 246/1992 Sb. on the protection of animals against cruelty and Decree 419/2012 on the protection of experimental animals (both were in accordance with the legislation of the European Parliament).

Harvested Capan-2 cells (5 × 10^6^) were administered subcutaneously in a mixture with BD Matrigel (VWR International; Prague, Czech Republic) into the abdominal right flank of outbred nude mice. When the tumors reached a diameter of ~6 mm, mice were randomly divided into control and experimental group (*n* = 4) and subjected to ketamine/xylasine narcosis. An aqueous dispersion of UCNP@PMVEMA and UCNP@PMVEMA-THPC-2 particles (100 mL; 1.5 mg/mL) was applied intratumorally. After 10 min, a 2 cm^2^ area was irradiated for 3 min using a Quanta System IG980 excitation laser (Medicom; Prague, Czech Republic) with a power of 1 W, a power density of 0.5 W/cm^2^, and an energy density of 90 J/cm^2^. Tumor volume and mouse weight were assessed twice a week, and mouse survival was followed for 30 days. 

## 3. Results and Discussions 

### 3.1. Synthesis and Characterization of UCNPs 

**Figure 2:**
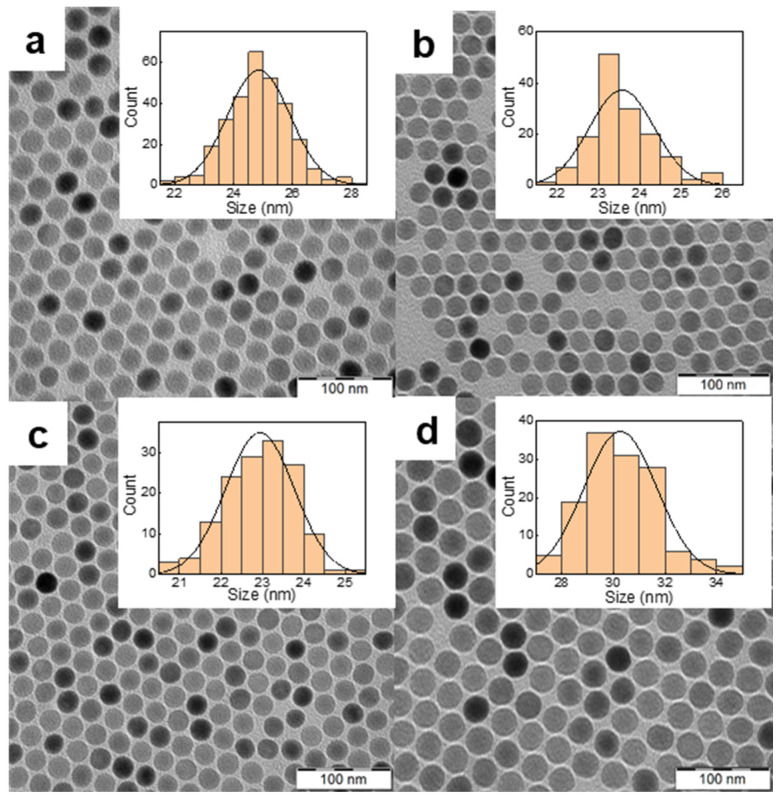
TEM micrographs of NaYF_4_:Yb^3+^,Er^3+^ particles doped with (**a**) 2, (**b**) 10 and (**c**) 15 mol.% of Er^3+^; (**d**) micrograph of NaYF_4_:Yb^3+^,Er^3+^,Fe^2+^ particles containing 15 mol.% Er^3+^ and 5 mol.% of Fe^2+^. The inset shows the corresponding particle size distributions.

Commonly used upconversion nanoparticles for PDT are characterized by strong green emission at 540 nm and negligible red emission at ~650 nm, where THPC absorbs light. The effective strategy to increase the emission intensity of NaYF_4_:Yb^3+^,Er^3+^ particles in the red region is their doping with various concentrations of lanthanide and iron ions [33,34]. The NaYF_4_:Yb^3+^,Er^3+^ nanoparticles were prepared by the high-temperature coprecipitation method using constant Yb^3+^ concentration (20 mol.%) and different Er^3+^ concentrations (2–15 mol.%). According to TEM, the particles were spherical and uniform in size, with *D*_n_ = 24 ± 1 nm and *Ð* = 1.01 (Figure 2a–c; Table S1). The monodispersity (*Ð* < 1.05) of the particles is crucial for their luminescence intensity, controlled size-dependent distribution in the organism, and bioelimination. The upconversion intensity (654 nm) of NaYF_4_:Yb^3+^(20 mol.%),Er^3+^(15 mol.%) particles (2 mg/mL) excited at 980 nm with a laser power of 2.11 W/cm^2^ slightly increased with increasing Er^3+^ concentration from 2 to 15 mol.% (Figure S2). Much more intense upconversion emission was observed for Fe^2+^-doped NaYF_4_:Yb^3+^,Er^3+^ particles, exhibiting 1.9- and 7.1-fold higher upconversion at 540 and 654 nm, respectively, compared to NaYF_4_:Yb^3+^(20 mol.%),Er^3+^(15 mol.%) particles (Figure S3). The incorporation of Fe^2+^ ions (5 mol.%) into the particles increased luminescence intensity, making the upconversion emission in the red region dominant and thus suitable for direct excitation of THPC as a PDT transducer. This was in agreement with the published ten- and eightfold enhancement of the red upconversion emission of NaYF_4_:Yb^3+^,Er^3+^ nanoparticles doped with Mn^2+^ and Fe^2+^ ions, respectively [34,35,36]. However, the exact effect of transition metal ions on the optical properties of upconversion nanoparticles is still difficult to understand and predict. In further experiments, NaYF_4_:Yb^3+^(20 mol.%),Er^3+^(15 mol.%),Fe(5 mol.%) particles, referred to as UCNPs, were used. 

According to TEM, the UCNPs were spherical in shape and monodisperse in size (*Ð* = 1.01; Figure 2; Table 1), which is important for their consistent physical, chemical, and biological properties and reproducibility of results. While the number-average diameter of NaYF_4_:Yb^3+^(20 mol.%),Er^3+^(15 mol.%) nanoparticles was 23 nm (Table S1), the particle size of UCNPs (doped with 5 mol.% Fe^2+^) increased to 30 nm (Table 1). This was in agreement with Fe^3+^-doped NaYF_4_:Yb^3+^,Er^3+^ particles with average diameters in the range of 23–47 nm [37]. Also, the size of Fe^2+^-doped NaYbF_4_:Er particles synthesized by the citrate-stabilized hydrothermal method was relatively large, 62–64 nm in diameter [34]. The polydispersity of UCNPs in water measured by DLS was small (*PD* = 0.11; Table 1), although the hydrodynamic diameter (*D*_h_ = 191 nm) was larger than *D*_n_, indicating a slight tendency to aggregate formation. Positively charged rare-earth ions on the UCNP surface were responsible for their positive ζ-potential (43 mV; Table 1). The TEM/EDX spectrum of the UCNPs showed major peaks of the Na, Y, F, and Yb atoms and a weaker Fe peak indicating the presence of Fe^2+^ in the particles; C and Cu peaks originated from the standard TEM supporting grid (Figure S4). The doping efficiency of Fe^2+^ ions was analyzed by inductively coupled plasma mass spectroscopy, which determined an Fe^2+^ concentration of 1 wt.% (Table S2). In addition, quantitative elemental analysis showed that the ratios of the rare-earth ion concentrations in the UCNPs agreed well with the stoichiometric ratios used for the synthesis (Table S2).

**Figure 3:**
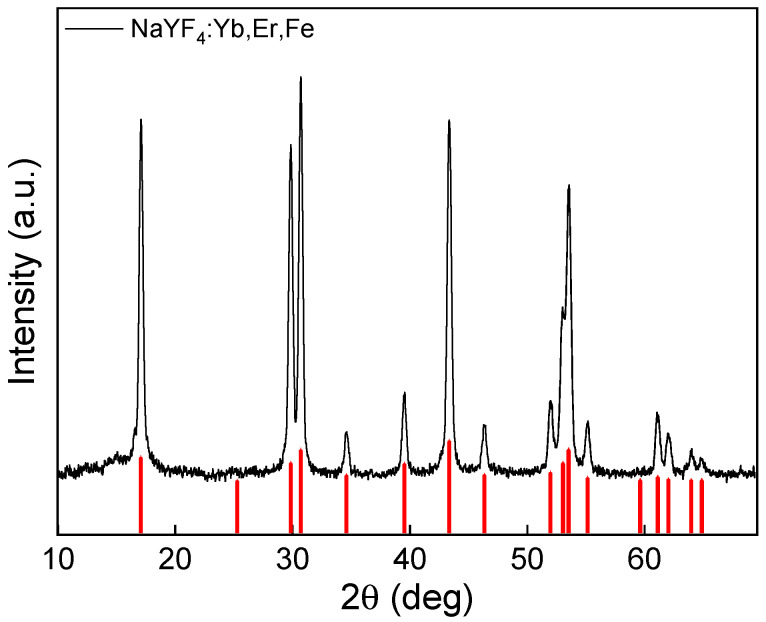
X-ray diffraction pattern of UCNPs. The red vertical lines indicate the standard hexagonal β-phase of NaYF_4_ (JCPDS card No. 28-1192).

The UCNP crystal structure and phase studied by an XRD technique demonstrated a highly crystalline structure with a calculated degree of crystallinity of 91.7% (Figure 3). The main intense peaks at *2θ =* 17.13, 29.91, 30.78, 43.46, and 53.67° corresponded to (100), (110), (101), (201), and (211) reflection planes, respectively; all peaks were indexed to the standard hexagonal β-phase of NaYF_4_ (JCPDS card no. 28-1192). The average crystallite sizes (*D*_XRD_) of UCNPs according to the Scherrer equation were 238.2 ± 7.6 Å, which agreed with TEM results. XRD of UCNPs demonstrated that dopant Fe^2+^ ions did not lead to the formation of other phases, in contrast to a previous report where Fe^3+^ ions caused the formation of mixed phases (α and β), which was probably due to a slightly different synthesis procedure [37]. 

### 3.2. Modification of UCNPs with PMVEMA 

Since the synthesized UCNPs are hydrophobic, their modification by a hydrophilic PMVEMA coating is necessary to ensure their functionality, colloidal and chemical stability in physiological media, and conjugation with THPC [27,38]. The presence of the PMVEMA containing carboxyl groups on the particle surface was accompanied by a change in the ζ-potential, which became negative (−29 mV; Table 1). At the same time, the hydrodynamic diameter of UCNP@PMVEMA particles in water increased to 237 nm (*PD* = 0.21). Despite this increase, the particles remained colloidally stable in a physiological saline solution (0.9% NaCl solution), the most commonly used medium in drug delivery systems that also replaces lost body fluids. The *D*_h_ of UCNP@PMVEMA particles in saline decreased to 106 nm at low polydispersity (*PD* = 0.12) and remained constant for 4 days with no signs of particle sedimentation. TEM micrographs of UCNP@PMVEMA nanoparticles showed their increased size (*D*_n_ = 39 nm; *Ð* = 1.01) with uniform coating thickness (~3 nm; Figure S5a; Table 1). According to TGA, the amount of PMVEMA on the particle surface was 5.7 wt.% (Figure S6a). The small weight loss (1.8 wt.%) during heating from RT to 200 °C was due to the evaporation of residual water; UCNP@PMVEMA and UCNPs still contained 1.2 wt.% of oleic acid. The ATR FTIR spectra of UCNPs (Figure S6b) exhibited characteristic bands of the residual oleic acid at 2927, 2861, 1657, 1574, and 1439 cm^−1^ attributed to ν_as_(CH_2_), ν_sym_(CH_2_), ν(C=C), ν_as_(COO−) and ν_sym_(COO−) stretching vibrations, respectively [38]. After the modification of UCNPs with PMVEMA, new peaks at 1708 and 1097 cm^−1^ were ascribed to the ν(C=O) and ν(C–O) stretching vibrations. Moreover, the intensities of the absorption peaks at 1572 and 1441 cm^−1^ ascribed to the asymmetric and symmetric stretching vibrations of the COOH groups of PMVEMA [27], respectively, were much higher compared to those of UCNPs, confirming the presence of polymers on the nanoparticle surface. 

It is well known that iron-containing nanoparticles can catalyze the Fenton reaction, producing damaging hydroxyl radicals [39,40]. Therefore, UCNP@PMVEMA nanoparticles were examined for the formation of the hydroxyl radicals generated by the autoxidation of Fe^2+^ ions. The generation of the hydroxyl radicals was monitored by the reaction of Fe^2+^ with H_2_O_2_, associated with the oxidative degradation of methylene blue and the decrease of the absorbance at 666 nm (Figure S7). The UV-Vis spectra measured in 2 min intervals and after 3 h of the incubation of particles in the solution of methylene blue in water and PBS did not show any decrease in the absorbance. In water, no significant decrease in absorbance was observed after the incubation of the particles for less than 24 h (Figure S7a). However, the generation of hydroxyl radicals was accelerated in PBS after 24 h (Figure S7b), because the UCNP@PMVEMA particles were more stable in saline solution than in water (Table 1). This allowed easy penetration of the solutions to the particle surface, catalyzing the Fenton reaction. PDT using UCNPs has the advantage of generating hydroxyl radicals that induce ferroptosis and apoptosis of tumor cells [41] and alleviate hypoxia [42]. 

**Figure 4:**
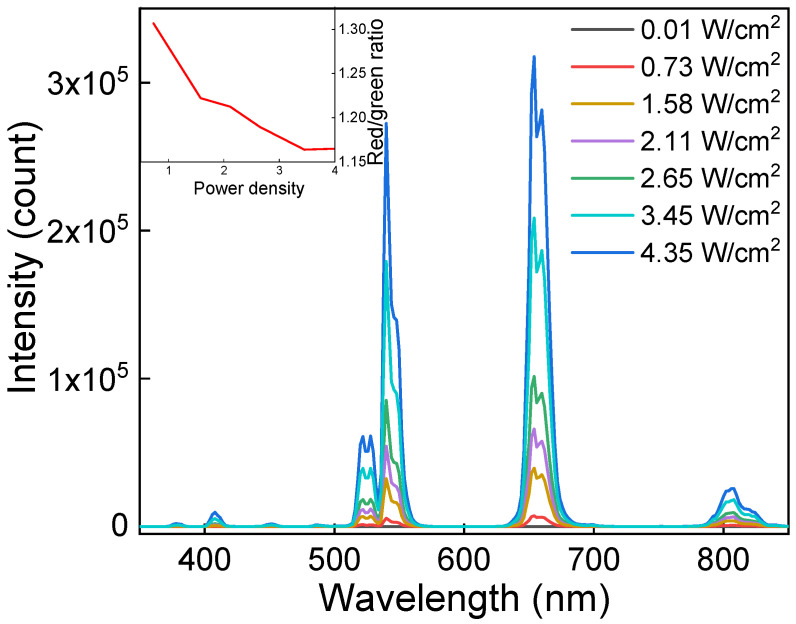
Upconversion photoluminescence emission spectra of UCNP@PMVEMA particles in water (1 mg/mL) excited at 980 nm with different power densities. The inset shows the dependence of red-to-green intensity ratio on laser power density.

The upconversion luminescence emission of UCNP@PMVEMA particles was measured under NIR excitation at 980 nm with a laser of different power densities (Figure 4). With increasing laser power, the intensity of both red and green Er^3+^ luminescence increased. The highest red-to-green emission of UCNPs was observed at low excitation intensity (0.73 W/cm^2^). It should be noted that the ratio was only slightly affected by the power density and did not depend on the particle concentration. The particles exhibited characteristic emissions at 409 nm (^2^H_9/2_→^4^I_15/2_), 525 nm (^2^H_11/2_→^4^I_15/2_), 542 nm (^4^S_3/2_→^4^I_15/2_), 654 nm (^4^F_9/2_→^4^I_15/2_), and 807 nm (^4^I_9/2_→^4^I_15/2_), which are typical of the Er^3+^ ion transitions in upconverting nanomaterials. The PMVEMA coating of UCNPs did not affect the emission intensity. 

### 3.3. Conjugation of THPC to UCNP@PMVEMA Particles 

Covalent attachment of THPC to the surface of UCNP@PMVEMA particles is a suitable approach to overcome the disadvantages associated with photosensitizer aggregation and release from the carrier, which would lead to a reduction in treatment efficacy. THPC was attached to UCNP@PMVEMA via carboxyl groups of PMVEMA, which were partially coordinated with the surface rare-earth ions of UCNP and partially available for conjugation with the hydroxyl groups of THPC via a solid ester linkage. 

**Figure 5:**
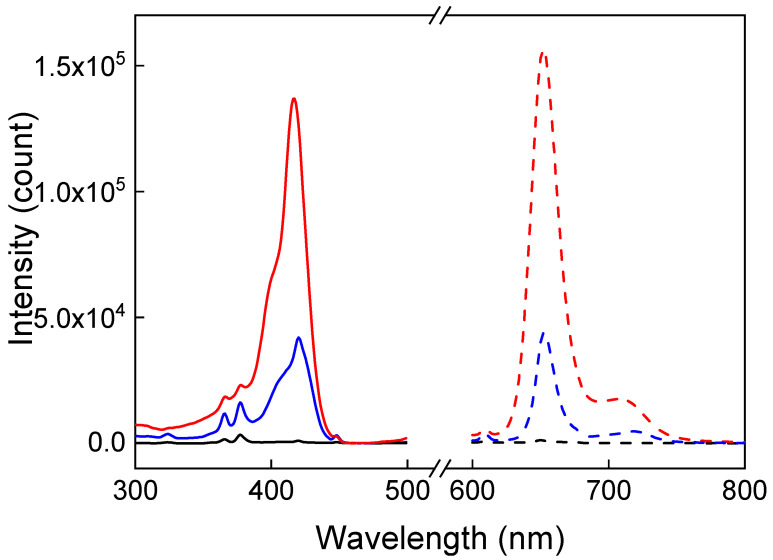
Excitation (solid line; λ_em_ = 652 nm) and emission (dashed line; λ_ex_ = 420 nm) spectra of UCNP@PMVEMA (black), UCNP@PMVEMA-THPC-1 (blue), and UCNP@PMVEMA-THPC-2 particles (red) in water (1 mg/mL).

The properties of the UCNP@PMVEMA-THPC particles were characterized by UV-Vis and fluorescence spectroscopy. It revealed a rather low THPC concentration on the surface of UCNP@PMVEMA-THPC-1 particles (0.4 μg/mg; Figure S8) obtained by the EDC- and DMAP-mediated Steglich reaction. However, when THPC was bound to UCNP@PMVEMA particles by NaOH-catalyzed esterification of THPC hydroxyl groups in DMSO [29], the resulting UCNP@PMVEMA-THPC-2 particles had a relatively high THPC content (1 μg/mg; Figure S8), as also confirmed by photoluminescence spectra (Figure 5). While the UCNP@PMVEMA particles did not show the characteristic excitation and emission peaks of THPC, its conjugation to the UCNP@PMVEMA particles resulted in luminescence at 652 nm and corresponding excitation at 420 nm. As expected, compared to UCNP@PMVEMA-THPC-1 particles with low THPC content, UCNP@PMVEMA-THPC-2 particles showed three times higher luminescence intensity. 

When UCNP@PMVEMA particles were conjugated with THPC, *D*_n_ and *Ð* remained almost unchanged (Figure S5b,c; Table 1). The hydrodynamic diameter of the UCNP@PMVEMA-THPC-2 particles in water increased slightly from 237 to 273 nm, and the polydispersity was moderate (*PD* = 0.33; Table 1). This partial particle aggregation was explained by the high content of hydrophobic THPC. In contrast, the *D*_h_ of UCNP@PMVEMA-THPC-1 nanoparticles with low THPC content decreased to 175 nm, and the size distribution was narrow (*PD* = 0.14; Table 1) due to the good stabilization efficiency of PMVEMA-THPC-1. The *ξ*-potential of UCNP@PMVEMA-THPC-1 and UCNP@PMVEMA-THPC-2 particles in water decreased to −52 and −33 mV, respectively (Table 1), as a result of the anionic nature of porphyrins. The observed shift of the *ξ*-potential of UCNP@PMVEMA-THPC particles provided further evidence for the successful conjugation of THPC to the UCNP@PMVEMA particle surface. In addition, all THPC-conjugated PMVEMA-coated nanoparticles were stable in saline solution; their *D*_h_ decreased to 150 ± 2 nm with *PD* < 0.2, confirming the relatively narrow size distribution. The absence of aggregation of UCNP@PMVEMA-THPC particles in water or physiological solution is important for future singlet oxygen generation and drug delivery. 

Singlet oxygen generation by THPC-conjugated UCNPs was detected spectrophotometrically via the bleaching of 1,3-diphenylisobenzofuran (DPBF) as a function of irradiation time at 980 nm excitation (Figure S9). DPBF was chosen as it has no absorbance in the NIR region used for laser irradiation. The addition of PMVEMA-coated UCNPs with/without THPC to DPBF in an ethanol/water mixture was monitored at 10 min intervals. The spectra of UCNP@PMVEMA nanoparticles demonstrated no effect after 120 min of exposure (Figure S9a). The decrease in DPBF absorption intensity at 415 nm for both THPC-1- and THPC-2-conjugated UCNP@PMVEMA particles demonstrated singlet oxygen generation due to the efficient energy transfer between UCNPs and THPC (Figure S9b,c). 

### 3.4. In Vitro Toxicity and Photodynamic Efficiency of UCNP@PMVEMA-THPC in Cells 

**Figure 6:**
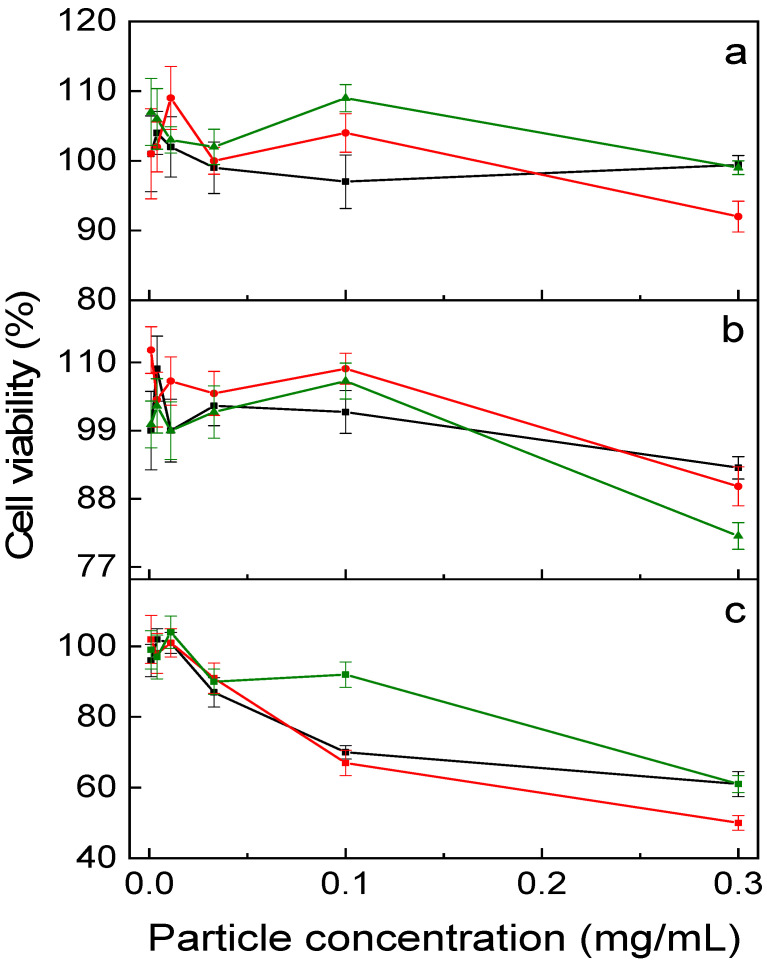
In vitro dark (no irradiation) cytotoxicity of UCNP@PMVEMA (black), UCNP@PMVEMA-THPC-1 (red), and UCNP@PMVEMA-THPC-2 particles (green) against (**a**) Capan-2, (**b**) Panc-01 and (**c**) PaTu-8902 pancreatic adenocarcinoma cell lines assessed by XTT assay.

Cytotoxicity testing of UCNP@PMVEMA-THPC nanoparticles prior to their use in PDT is necessary to ensure safety and avoid side effects in the absence of 980 nm irradiation. Thus, to determine the dark toxicity, UCNP@PMVEMA and UCNP@PMVEMA-THPC-1 and -2 particles were tested in vitro on three human pancreatic adenocarcinoma cell lines (Capan-2, PANC-01, and PA-TU-8902) using the XTT assay after 48 h of incubation (Figure 6). In the case of Capan-2 and PANC-01 cell lines, no decrease in viability was observed after incubation with all nanoparticles used in the concentration range of 0–0.3 mg/mL. Consequently, PMVEMA-coated UCNPs with/without THPC can be considered nontoxic to these cells. However, in the case of PA-TU-8902 cells, all particles tested were weakly toxic at the highest concentration (0.3 mg/mL), with cell viability ranging from 80–50%; UCNP@PMVEMA-THPC-2 had the lowest impact. This high concentration corresponds to a hypothetical dose of 1.5 g of particles in whole blood, which seems abnormally high. The data were also comparable to those previously reported for THPC-containing UCNPs, which potentially qualifies them for PDT [26].

**Figure 7:**
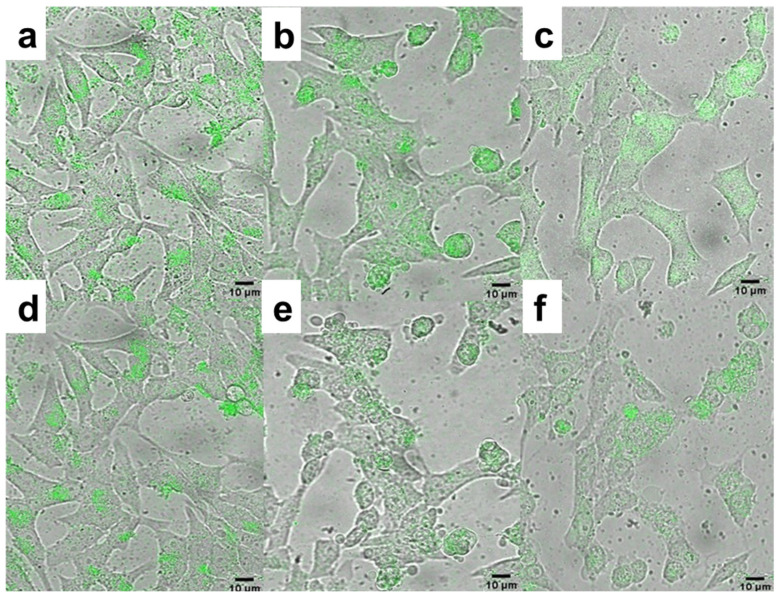
Overlays of bright field micrographs of (**a**,**d**) UCNP@PMVEMA, (**b**,**e**) UCNP@PMVEMA-THPC-1, and (**c**,**f**) UCNP@PMVEMA-THPC-2 particles (green) in rat insulinoma INS-1E cells and upconversion photoluminescence after (**a**–**c**) 0 and (**d**–**f**) 30 min of excitation at 980 nm and detection at 500−700 nm. Scale bar 10 μm.

In the next experiments, the photodynamic effect of THPC-conjugated PMVEMA-coated UCNPs on INS-1E rat insulinoma cells as a pancreatic β-cell tumor model was examined. Cells were incubated with an aqueous dispersion of UCNP@PMVEMA-THPC-1 and -2 nanoparticles and UCNP@PMVEMA as a control and irradiated with a 980 nm laser (Figure 7). THPC on the surface of the particles increased their intracellular accumulation as an overlay of bright field micrographs showed the particle distribution in the cell cytoplasm (Figure 7a–c). The difference in emission spectra of internalized particles and cell autofluorescence confirmed the labeling of cells with nanoparticles (Figure S10). After 30 min of laser exposure, UCNP@PMVEMA-THPC conjugates were able to convert oxygen into ^1^O_2_ in cells, leading to their destruction and demonstrating PDT activity (Figure 7e,f). In addition, cells both without particles and incubated with UCNP@PMVEMA particles were not affected by laser irradiation at 980 nm for 30 min (Figure 7d). The death of tumor cells incubated with non-toxic UCNP@PMVEMA-THPC particles irradiated with NIR thus demonstrated efficient ROS generation and their applicability in photodynamic therapy. The UCNP@PMVEMA-THPC-2 particles with the highest THPC content that did not affect cell viability were then selected for in vivo photodynamic treatment. 

### 3.5. In Vivo PDT 

**Figure 8:**
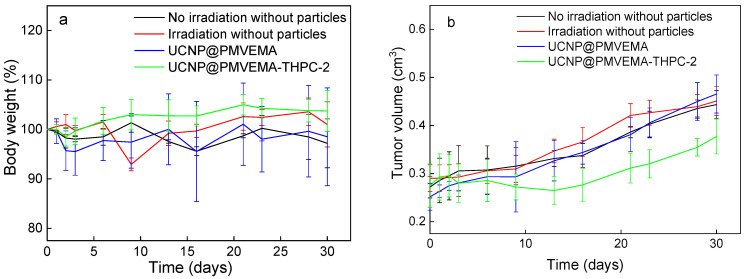
In vivo photodynamic therapy with UCNP@PMVEMA-THPC-2 particles. Time dependence of (**a**) nu/nu mice weight and (**b**) tumor growth (*n* = 4); the mice with subcutaneously growing Capan-2 pancreatic tumors were intratumorally (i.t.) injected with 100 µL of PBS (two control groups) or UCNP@PMVEMA and UCNP@PMVEMA-THPC-2 particles and irradiated with a 980-nm laser 10 min after administration. No evidence of systemic toxicity was documented. Controls: untreated mice with i.t.-injected PBS without and with irradiation. Statistical significance between UCNP@PMVEMA-THPC-2 and other three groups *p* < 0.05.

**Figure 9:**
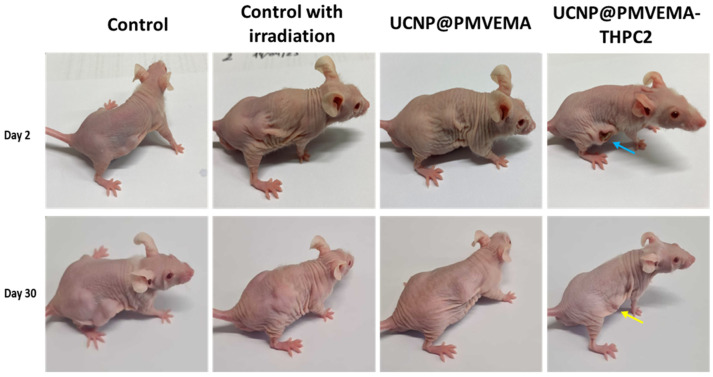
Nu/nu mice with growing Capan-2 human pancreatic adenocarcinoma two (upper line) and thirty days (lower line) after 980-nm NIR-induced PDT. Blue arrow—necrosis in the group treated with UCNP@PMVEMA-THPC-2 particles; yellow arrow—the scar after healed tumor. Controls: untreated mice with i.t.-injected PBS without and with irradiation.

The efficacy of UCNP@PMVEMA-THPC-2 conjugates for PDT after irradiation with 980 nm NIR light was tested in vivo in a pilot study on human pancreatic adenocarcinoma (Capan-2) growing subcutaneously in athymic nude mice. Mice were intratumorally administered with either PBS (control group), UCNP@PMVEMA, or UCNP@PMVEMA-THPC-2 particles, and then exposed to a 980 nm laser for 3 min at a power density of 0.5 W/cm^2^ (except the negative control group). It should be noted that the NIR laser power used in this study was excited at a power density of <0.7 W/cm^2^, which is the conservative limit set for safe exposure of human skin to 980 nm light [43]. In order to detect any signs of toxicity of the tested UCNPs, changes in the average weight of the mice and the volume of the extracted tumors were monitored in all group members twice a week (Figure 8). However, there was no significant difference in body weight between the groups (Figure 8a), suggesting that no side effects occurred during the treatment period; the same was observed for upconversion nanoparticles containing chlorin e6 [44]. On the first or second day after irradiation, all four mice in the UCNP@ PMVEMA-THPC-2 particle-treated group developed extensive necrosis (Figure 9), whereas no necrosis was observed in the control and UCNP@PMVEMA-containing groups. No mouse was completely cured in our setting. Over a period of thirty days, tumors were measured twice weekly using calipers, and tumor volumes were calculated as follows: *a b*^2^/2 (*a*—longer diameter; *b*—shorter diameter). A comparison of the two control groups (PBS and no irradiation vs. PBS and irradiation) and the UCNP@PMVEMA group revealed no significant difference in tumor growth. Their volume at the end of the pilot study (day 30) ranged from 0.45 to 0.47 cm^3^. However, the UCNP@PMVEMA-THPC-2 group showed statistically significant suppression of tumor growth rate, with tumor volume ranging from 0.35 cm^3^ at day 30 (Figure 8b). Thus, the potential of THPC-conjugated PMVEMA-coated UCNPs in the treatment of pancreatic adenocarcinoma was confirmed. Nevertheless, further improvement of the NIR-induced UCNP-based PDT requires optimization of PDT treatment (e.g., power density and exposure time). In addition, further research on these promising nanoparticles will focus on their biocompatibility and biodistribution after different routes of administration (intravenous, intraperitoneal, or intratumoral) and testing their efficacy against other tumor types.

So far, a wide range of different phototherapeutic agents based on PS-conjugated upconversion nanoparticles have been tested for NIR-induced PDT, with the main objective of demonstrating cellular uptake and cell death after NIR irradiation [2,11,45]. The first in vivo application was performed by intratumoral injection of NaYF_4_:Yb^3+^,Er^3+^@poly(ethylene glycol) particles and non-covalently loaded chlorin-e6 (Ce6) into 4T1 breast tumor mice [22]. PDT treatment with 980 nm irradiation (0.5 W/cm^2^; 30 min) significantly reduced the tumor growth rate in the mice. The same effect after NIR irradiation (5 min; 0.6 W/cm^2^) was observed in nude mice bearing U87MG tumors injected intravenously with Ce6-loaded NaYF_4_:Yb^3+^,Er^3+^@NaGdF_4_@poly(ethylene glycol)-phospholipid particles [46]. S180 sarcoma tumor-bearing mice treated with zinc phthalocyanine-loaded chitosan-modified NaYF_4_:Yb^3+^,Er^3+^ particles, which were irradiated twice (3 h and 24 h post-injection) with a 980 nm laser (0.4 W/cm^2^; 15 min), showed only a small increase in tumor volume and a high survival rate [47]. Injection of NaYF_4_:Yb^3+^,Er^3+^@CaF_2_@poly(acrylic acid)-hydrazide particles covalently functionalized with 5-aminolevulinic acid into mice significantly reduced their tumor size after irradiation with a 980 nm laser (0.5 W/cm^2^) for 40 min [48]. An in vivo PDT study on mice bearing CT-26wt tumors showed that tumor growth was reduced after intratumoral injection of tetraphenylporphine-loaded NaYF_4_:Yb^3+^,Er^3+^@phosphatidylcholine-folic acid particles and irradiation at 980 nm (1 W/cm^2^; 30 min) [49]. Similarly, many organic photosensitizers such as methylene blue [50], Rose Bengal [51], and pyropheophorbide-a methyl ester [52] integrated with upconversion nanoparticles for NIR-activated PDT (0.15–1 W/cm^2^; up to 40 min) significantly inhibited tumor growth. Their results of NIR-induced PDT are consistent with our work. Only one attempt to investigate THPC and upconversion nanoparticles in vivo has been reported [53]. Encapsulation of THPC, IR-780 dye in NaYF_4_:Yb^3+^,Er^3+^@PEG particles provided promising treatment of glioblastoma under combined 980 nm (0.8 W/cm^2^)/808 nm (0.36 W/cm^2^) laser irradiation for 5 min. Considering the above works, the present report demonstrates the prospects PDT treatment of cancer in vivo using THPC-conjugated UCNP@PMVEMA particles at moderate NIR irradiation (0.5 W/cm^2^) for 3 min.

## 4. Conclusions 

Although a number of UCNP-based PDT platforms have been described, only a few of them use clinically approved photosensitizers. Meanwhile, the use of clinically approved PSs will greatly accelerate the approval process for these PDT systems. To the best of our knowledge, there are so far no reports on Fe^2+^-doped UCNPs with chemically bound THPC for NIR-induced PDT. Hence, upconversion NaYF_4_:Yb^3+^,Er^3+^,Fe^2+^ nanoparticles were prepared by thermal decomposition of the corresponding chlorides. The upconversion emission of these particles excited at 980 nm was dependent on the Er^3+^ concentration. The incorporation of Fe^2+^ ions (5 mol.%) into the particles increased the fluorescence intensity, making the upconversion emission in the red region dominant, and allowed producing hydroxyl radicals to reach more effective PDT. Thanks to the hydrophilic PMVEMA coating, the nanoparticles were highly colloidally stable in water and physiological saline solution. Covalent binding of THPC to the UCNP@PMVEMA particle surface provided colloidally stable conjugates, which enabled the generation of ROS, as demonstrated by the DPBF assay. The esterification of THPC hydroxyl groups introduced a higher THPC content in UCNP@PMVEMA particles than in the case of the Steglich reaction. The in vitro assay on pancreatic cancer cells demonstrated non-cytotoxic behavior of the UCNP@PMVEMA-THPC particles and significant cell destruction after excitation with a 980 nm laser. A pilot in vivo PDT experiment demonstrated promising efficacy in the treatment of human pancreatic adenocarcinoma growing subcutaneously in athymic mice. Irradiation with a 980 nm laser 10 min after intratumoral administration of UCNP@PMVEMA-THPC particles significantly hindered tumor growth. Even though complete remission was not observed, all the animals manifested the efficacy of PDT by developing necrosis in all irradiated tumors. This study highlights the great potential of the developed temoporfin-containing upconversion nanoconjugates as an innovative PDT agent for future translational research on NIR-induced photodynamic therapy.

## Figures and Tables

**Table 1:** Characterization of the nanoparticles.

Particles	*D*_n_ (nm)	*Ð*	*D*_h_ (nm) Water	*PD* Water	*D*_h_ (nm) Saline	*PD* Saline	*ξ*-Potential (mV)
UCNPs	30	1.01	191	0.11	1433	0.38	43
UCNP@PMVEMA	39	1.01	237	0.21	106	0.12	−29
UCNP@PMVEMA-THPC-1	38	1.01	175	0.14	148	0.11	−52
UCNP@PMVEMA-THPC-2	36	1.01	273	0.33	152	0.16	−33

*D*_n_—number-average diameter (TEM), *Ð*—dispersity (TEM), *D*_h_—hydrodynamic diameter (DLS), *PD*—polydispersity (DLS).

## Data Availability

All relevant data are contained within the article. The original contributions presented in the study are included in the article/supplementary material, further inquiries can be directed to the corresponding authors.

## Supplementary Materials

The following supporting information can be downloaded at: https://www.mdpi.com/article/10.3390/pharmaceutics15122694/s1, Table S1. Characterization of the nanoparticles with different Er content; Table S2. Concentrations of ions in UCNPs determined by atomic absorption and inductively coupled plasma mass spectroscopy; Figure S1. Chemical structure of (a) poly(methyl vinyl ether-*alt*-maleic acid) (PMVEMA) and (b) 5,10,15,20-tetra(m-hydroxyphenyl)chlorin (THPC; temoporfin; Foscan^®^). Figure S2. Normalized upconversion photoluminescence emission spectra of NaYF_4_:Yb (20 mol.%),Er nanoparticles with different Er content excited at 980 nm with a power density of 2.11 W/cm^2^; Figure S3. Upconversion photoluminescence emission spectra of NaYF_4_:Yb(20 mol%),Er(15 mol%) aqueous dispersions (2 mg/mL) codoped with Fe^2+^ excited at 980 nm with a power density of 2.11 W/cm^2^; Figure S4. TEM/EDX analysis of UCNPs; Figure S5. TEM micrograph of (a) UCNP@PVMEMA, (b) UCNP@PVMEMA-THPC-1, and (c) UCNP@PVMEMA-THPC-2 particles; Figure S6. (a) TGA thermograms and (b) ATR FTIR spectra of UCNPs and UCNP@PMVEMA nanoparticles; Figure S7. UV-Vis spectra of methylene blue degradation in (a) water and (b) PBS caused UCNP@PVMEMA particles and H_2_O_2_; Figure S8. UV-Vis absorption spectra of UCNP@PVMEMA, UCNP@PVMEMA-THPC-1, and UCNP@PVMEMA-THPC-2; Figure S9. UV-Vis spectra of DPBF in ethanol/H_2_O (50/50; *v*/*v*) containing (a) UCNP@PMVEMA, (b) UCNP@PVMEMA-THPC-1 and (c) UCNP@PVMEMA-THPC-2 particles versus irradiation time (min) at 980 nm excitation with a power density of 2.11 W/cm^2^. The time-dependent decrease in the absorbance of DPBF is evidence of ^1^O_2_ generation; Figure S10. Normalized upconversion photoluminescence emission spectra of UCNP@PVMEMA, UCNP@PVMEMA-THPC-1, and UCNP@PVMEMA-THPC-2 localized in the INS-1E cells and cell autofluorescence without particles excited at 980 nm.
