# Supplementary material for: Temoporfin-Conjugated Upconversion Nanoparticles for NIR-Induced Photodynamic Therapy: Studies with Pancreatic Adenocarcinoma Cells In Vitro and In Vivo
Source: Pharmaceutics. 2023 Nov 28;15(12):2694. doi: 10.3390/pharmaceutics15122694 (PMC10748036; doi:10.3390/pharmaceutics15122694)
Supplement: Supplementary file 1 [file pharmaceutics-15-02694-s001.zip › pharmaceutics-2715367-supplementary.pdf]

Supporting Information

NIR-induced photodynamic therapy of pancreatic tumor using temoporfin-conjugated poly(methyl vinyl ether-*alt*-maleic acid)-coated upconversion nanoparticles in animal model

Table S1. Characterization of the nanoparticles with different Er content.

Particles	D_n (nm)	$Đ$
$NaYF_4$:Yb,Er(2 mol.%)	25±1	1.01
$NaYF_4$:Yb,Er(10 mol.%)	24±1	1.01
$NaYF_4$:Yb,Er(15 mol.%)	23±1	1.01

D_n – number-average particle diameter (TEM); $Đ$ – dispersity (TEM).

Table S2. Concentrations of ions in UCNPs determined by atomic absorption and inductively coupled plasma mass spectroscopy.

	Concentrations	
	(wt.%)[a]	(M)
$[Na^{3+}]$[1]	12.1	5.28
$[Y^{3+}]$[2]	26.9	3.03
$[Yb^{3+}]$[2]	13.4	0.80
$[Er^{3+}]$[2]	18.6	1.07
$[Fe^{2+}]$[1]	1.0	0.18

[a] Percentage represents relative weight content of the specific ions a in the particles according to [1] atomic absorption and [2] inductively coupled plasma mass spectroscopy.

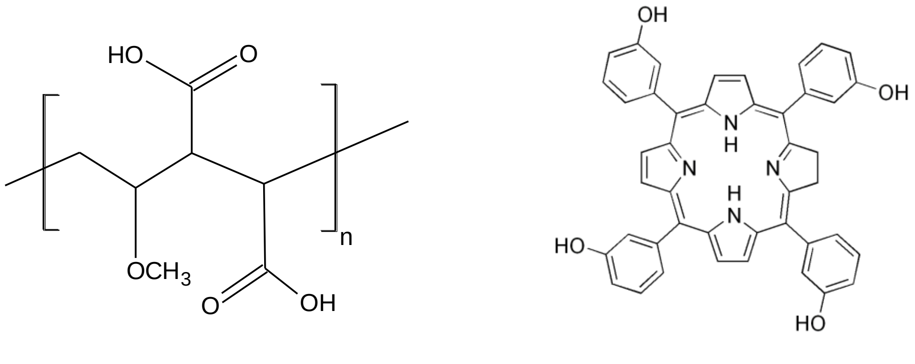

a b

Figure S1. Chemical structure of (a) poly(methyl vinyl ether-*alt*-maleic acid) (PMVEMA) and (b) 5,10,15,20-tetra(m-hydroxyphenyl)chlorin (THPC; temoporfin; Foscan®).

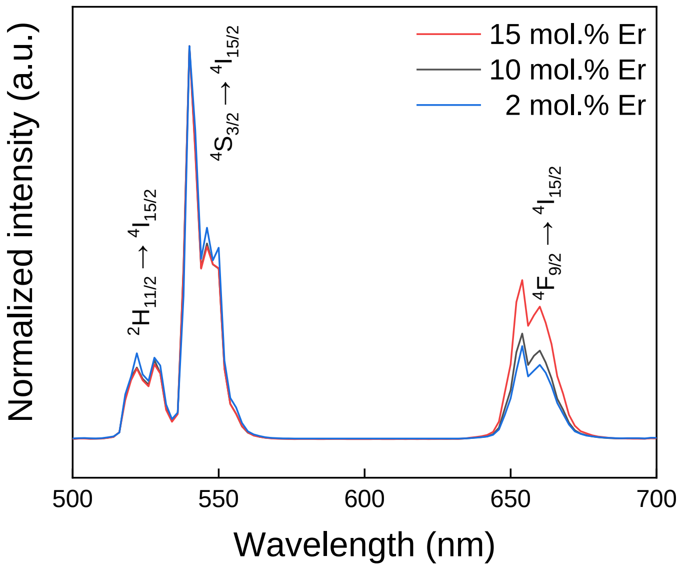

Figure S2. Normalized upconversion photoluminescence emission spectra of $NaYF_4$:Yb (20 mol.%),Er nanoparticles with different Er content excited at 980 nm with a power density of 2.11 W/cm^2.

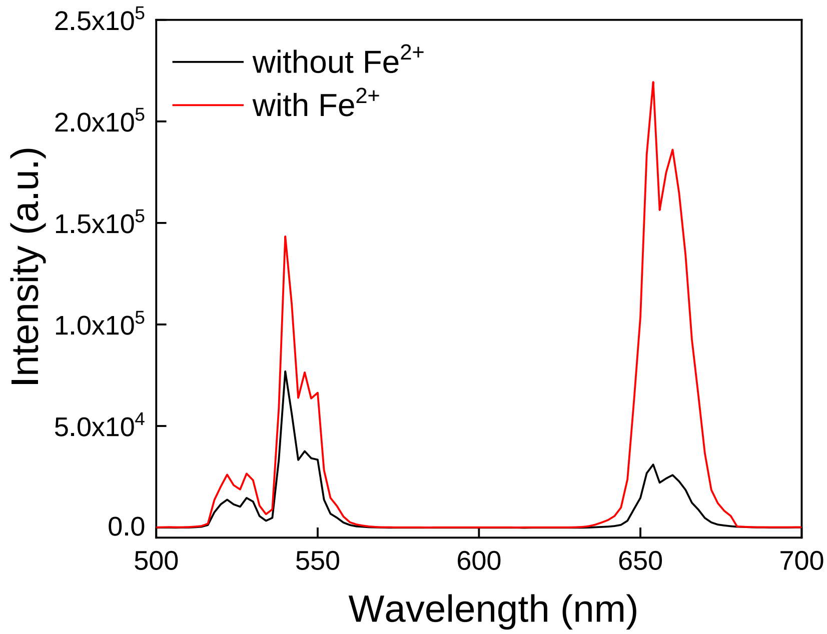

Figure S3. Upconversion photoluminescence emission spectra of $NaYF_4$:Yb(20 mol%),Er(15 mol%) aqueous dispersions (2 mg/ml) codoped with Fe^{2+} excited at 980 nm with a power density of 2.11 W/cm^2.

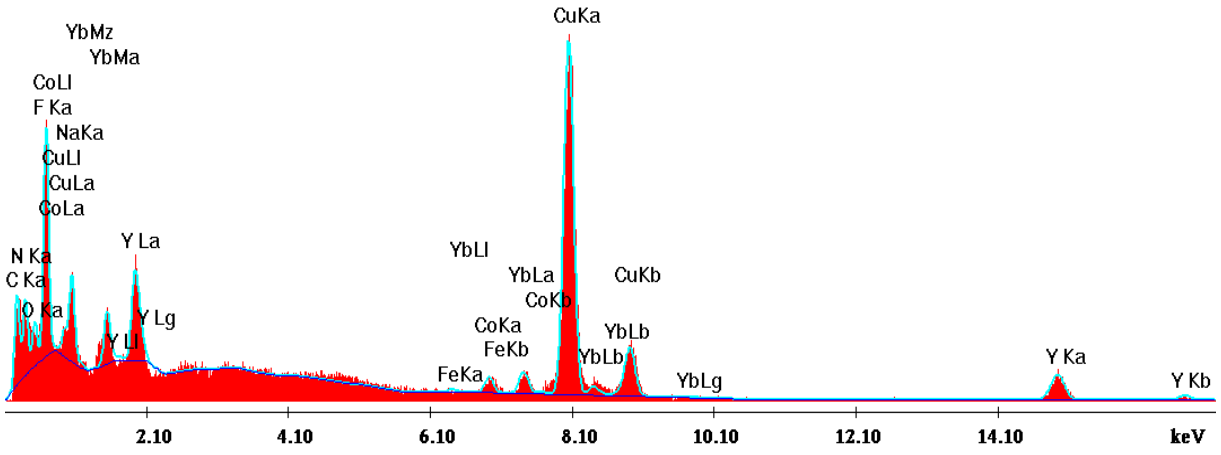

Figure S4. TEM/EDX analysis of UCNPs.

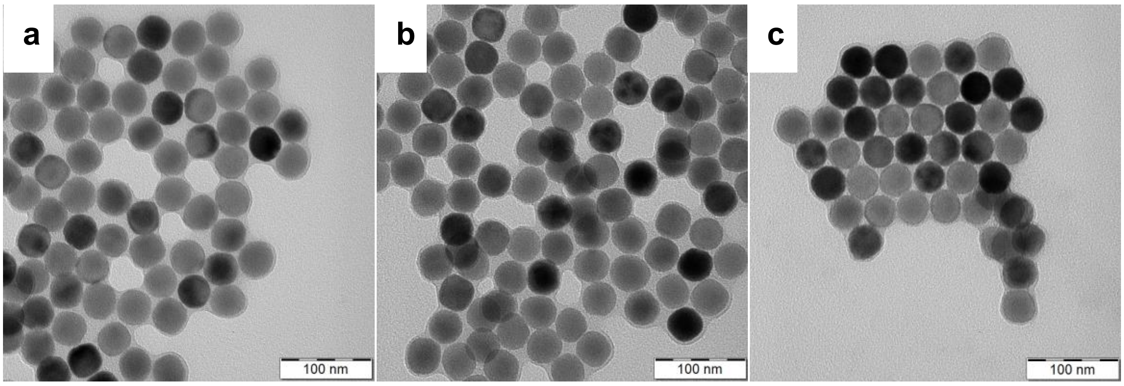

Figure S5. TEM micrograph of (a) UCNP@PVMEMA, (b) UCNP@PVMEMA-THPC-1 and (c) UCNP@PVMEMA-THPC-2 particles.

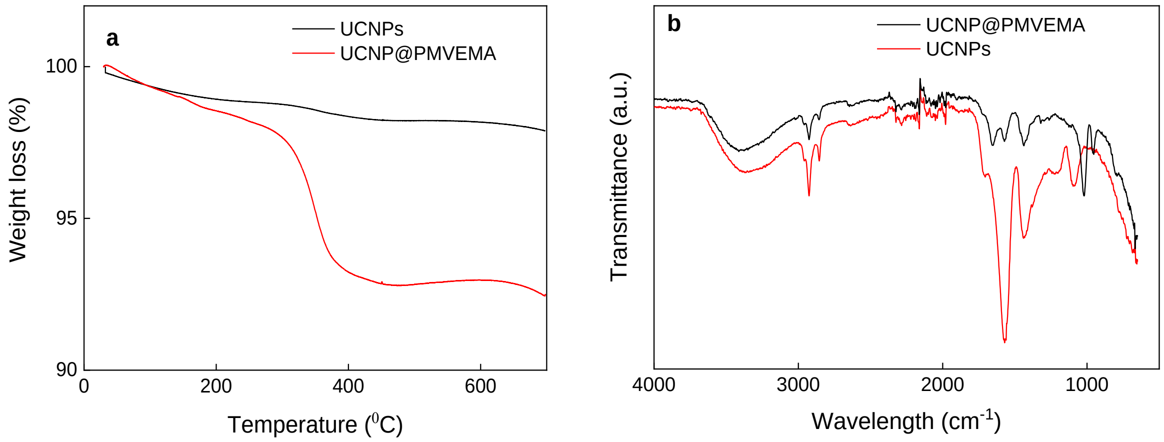

Figure S6. (a) TGA thermograms and (b) ATR FTIR spectra of UCNPs and UCNP@PMVEMA nanoparticles.

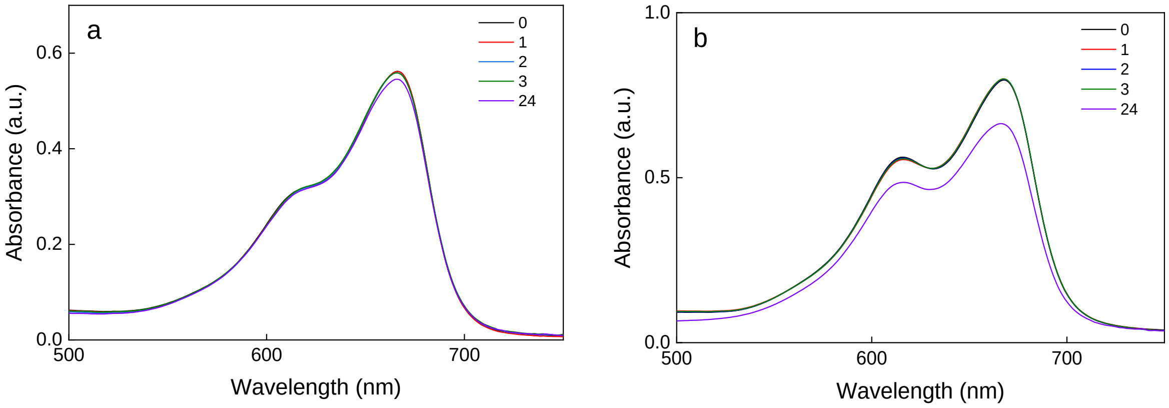

Figure S7. UV-Vis spectra of methylene blue degrading in (a) water and (b) PBS in the presence of UCNP@PVMEMA particles and H_2O_2.

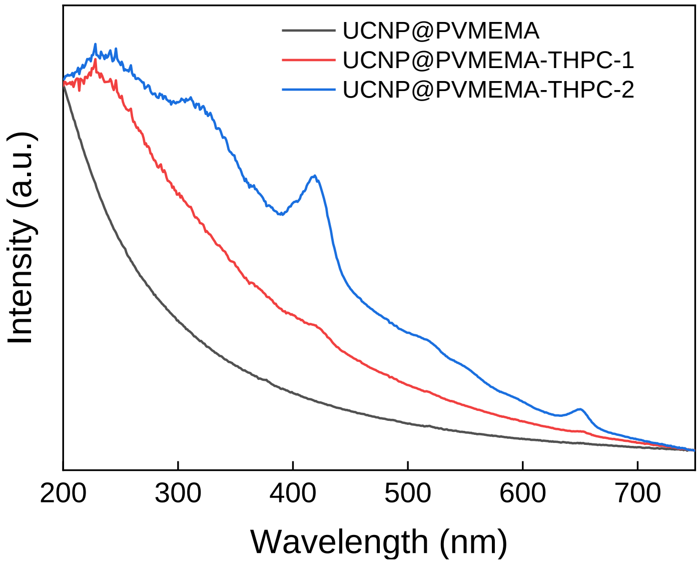

Figure S8. UV-Vis absorption spectra of UCNP@PVMEMA, UCNP@PVMEMA-THPC-1 and UCNP@PVMEMA-THPC-2.

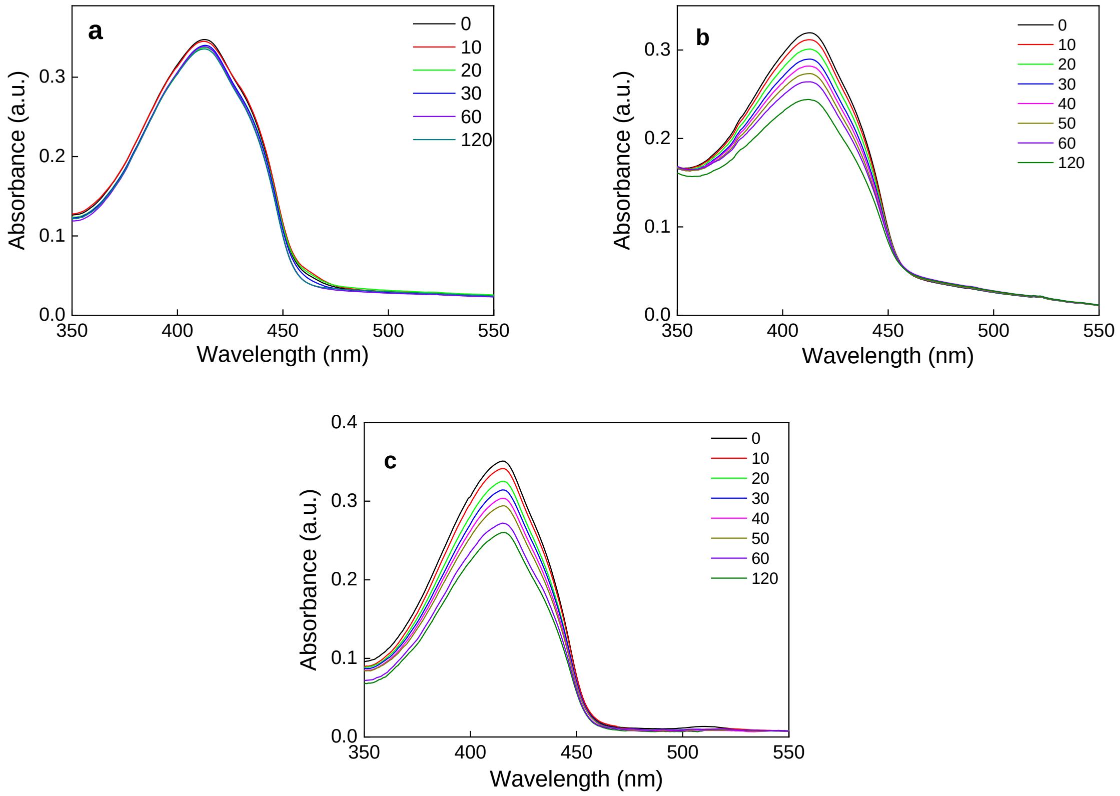

Figure S9. UV-Vis spectra of DPBF in ethanol/H_2O (50/50; v/v) containing (a) UCNP@PVMEMA, (b) UCNP@PVMEMA-THPC-1 and (c) UCNP@PVMEMA-THPC-2 particles versus irradiation time (min) at 980 nm excitation with a power density of 2.11 W/cm^2. The time-dependent decrease in the absorbance of DPBF is evidence of $^1O_2$ generation.

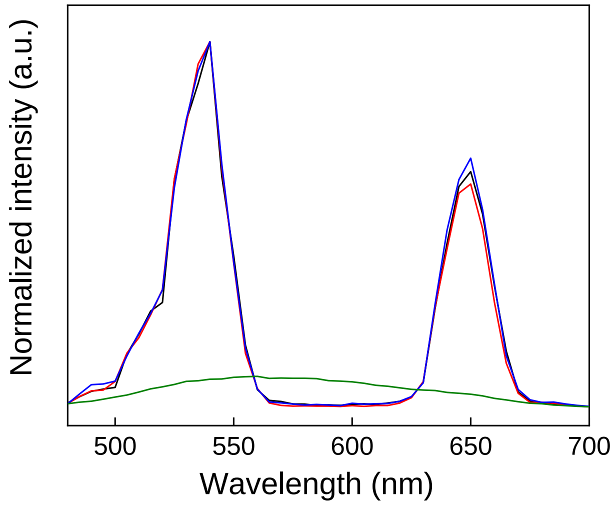

Figure S10. Normalized upconversion photoluminescence emission spectra of UCNP@PVMEMA (black), UCNP@PVMEMA-THPC-1 (red) and UCNP@PVMEMA-THPC-2 (blue) localized in the INS-1E cells and cell autofluorescence without particles (green) excited at 980 nm.
